# Imaging features and percutaneous endoscopic interlaminar decompression of vacuum disc phenomenon-induced lumbosacral radiculopathy: case report

**DOI:** 10.3389/fsurg.2025.1595166

**Published:** 2025-07-17

**Authors:** Longxiao Wu, Saifei Meng, Peng Li, Chunlei Liu

**Affiliations:** Department of Spinal Surgery, Qingyuan Hospital Affiliated to Guangzhou Medical University, Qingyuan People's Hospital, Qingyuan, Guangdong, China

**Keywords:** vacuum disc phenomenon, multimodal imaging, lumbosacral radiculopathy, percutaneous endoscopic interlaminar discectomy (PEID), minimally invasive spine surgery

## Abstract

The vacuum disc phenomenon (VDP), characterized by gas accumulation within degenerated intervertebral discs, is a radiographic hallmark of advanced spinal degeneration. Although this phenomenon is rare, VDP may also rarely present as radiculopathy due to the compression of nerve structures by dynamic gas migration—which is different from typical intervertebral disc protrusion. This phenomenon predominantly affects elderly populations, with computed tomography (CT) imaging serving as the gold standard for detecting hypodense gas pockets and delineating their spatial relationship to nerve roots. Unlike mechanical compression from disc fragments, gas-induced symptoms are uniquely refractory to conservative therapies, necessitating targeted surgical strategies. This report details a 72-year-old female with acute L5 radiculopathy secondary to multilevel VDP, where percutaneous endoscopic interlaminar decompression achieved immediate symptom resolution through precise gas evacuation. The case underscores the critical interplay between imaging interpretation and minimally invasive intervention in addressing this rare yet debilitating complication of spinal degeneration, while advocating for standardized protocols to optimize patient selection and outcomes.

## 1 Introduction

The vacuum disc phenomenon (VDP), characterized by intravertebral gas accumulation, is commonly observed in degenerative spinal diseases and is typically asymptomatic (1). However, when gas compresses nerve roots or the dural sac, it may lead to low back pain and sciatica, mimicking symptoms of lumbar disc herniation, though such cases are rare (2, 3). Radiculopathy secondary to VDP is often refractory and poorly responsive to conservative management (4). With advancements in minimally invasive techniques, percutaneous Endoscopic Interlaminar Discectomy (PEID) has emerged as a preferred treatment for symptomatic VDP, offering advantages of minimal trauma and rapid recovery (5). This report presents a 72-year-old female with lumbosacral radiculopathy caused by gas compression at L4–5 and L5–S1 levels. Imaging studies [computed tomography (CT) and magnetic resonance imaging (MRI)] confirmed gas accumulation compressing the right L5 nerve root. PEID successfully evacuated the gas, resulting in immediate symptom relief. This case highlights the efficacy of endoscopic decompression in managing VDP-induced radiculopathy.

## 2 Case report

A 72-year-old female was admitted on January 15, 2025, with a 10-day history of low back pain radiating to the right lower limb after walking. The pain was described as a persistent dull ache aggravated by movement. The local hospital administered nonsteroidal anti-inflammatory drugs for analgesic treatment for one week, with unsatisfactory results. Physical examination revealed an antalgic gait, tenderness over the L4/5 spinous process and right paravertebral region, right first toe dorsiflexion strength of Grade IV, hypoesthesia on the right foot dorsum, and a positive right straight leg raise test at 40°. Bilateral knee and ankle reflexes were normal, with no clonus or pathological reflexes.

**Figure 1:**
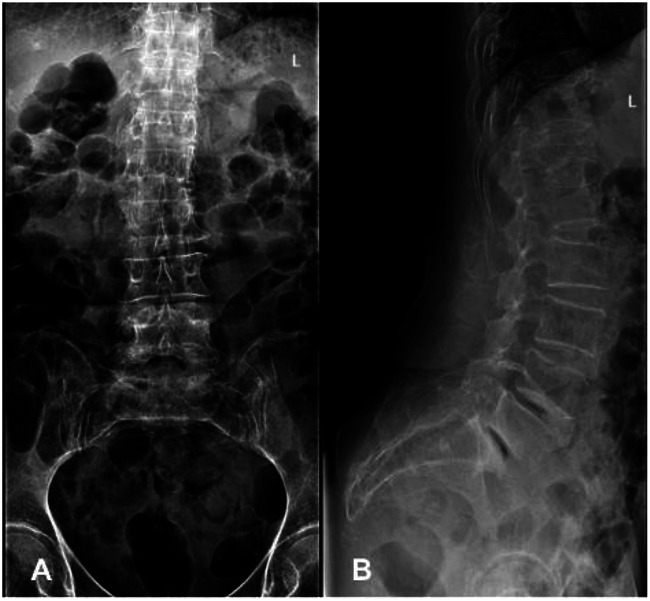
Preoperative anteroposterior and lateral lumbar radiographs. **(A)** Anteroposterior view demonstrates a radiolucent shadow at the L5/S1 interlaminar space, suggestive of gas accumulation. **(B)** Lateral view reveals the vacuum disc phenomenon at L4/5 and L5/S1 levels, accompanied by L5 spondylolysis.

**Figure 2:**
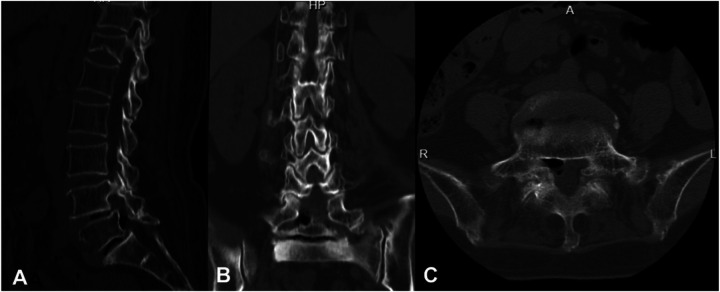
Preoperative lumbar CT scans. **(A)** Sagittal view demonstrates the vacuum disc phenomenon at L4/5 and L5/S1 level, with gas accumulation along the posterior margin of the L5 vertebral body. **(B)** Coronal view reveals gas within the right L5 neural foramen. **(C)** Axial view highlights gas accumulation in the right L5 lateral recess.

**Figure 3:**
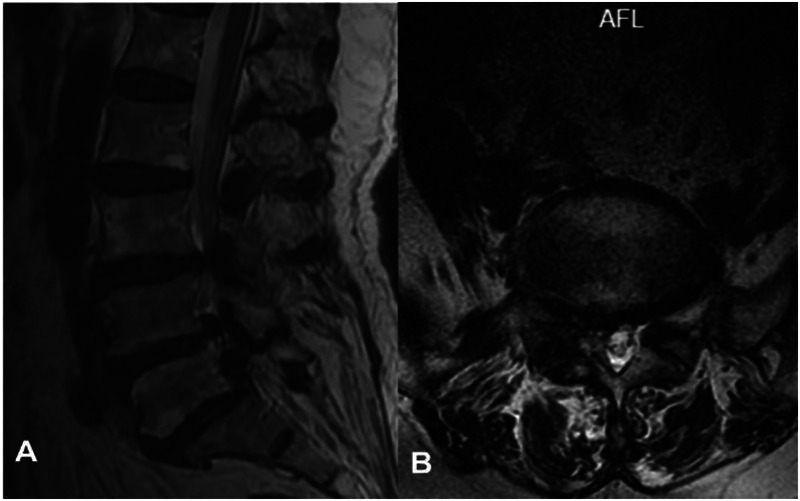
Preoperative lumbar MRI. **(A)** Sagittal view shows hypointense signals along the posterior margins of the L4/5 and L5/S1 vertebral bodies, consistent with gas accumulation. **(B)** Axial view demonstrates heterogeneous signal intensity in the right L5 lateral recess.

Imaging studies included lumbar radiographs showing narrowing of the L4/5 and L5/S1 intervertebral spaces, VDP, and L5 spondylolysis (Figures 1A,B). Lumbar CT confirmed gas accumulation at L4/5 and L5/S1 levels, with gas extending to compress the right L5 nerve root at the L5/S1 foramen (Figures 2A–C). Lumbar MRI T2-weighted imaging (T2WI) revealed narrowing of the intervertebral spaces at L4/5 and L5/S1, with ovoid hypointense areas within the intervertebral discs and at the right posterior margin of the L5 vertebral body. Heterogeneously hyperintense signal was observed in the right lateral recess of L5/S1, indicative of degenerative changes in the nucleus pulposus (Figures 3A,B).

**Figure 4:**
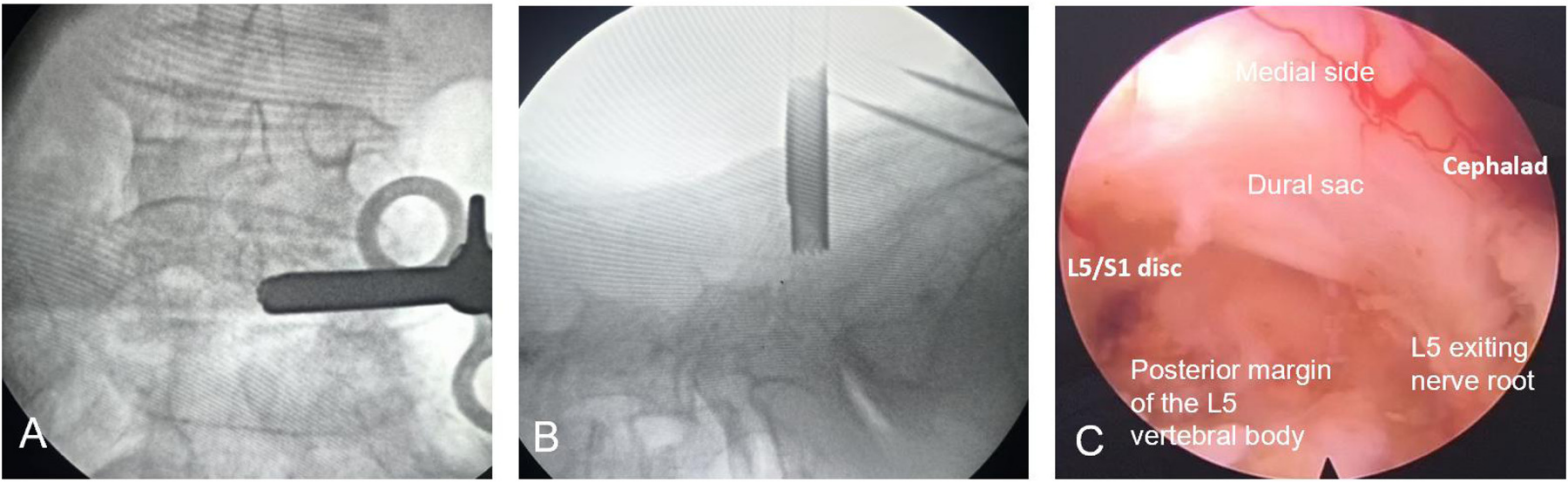
Intraoperative endoscopic procedure. **(A,B)** Intraoperative fluoroscopic guidance. **(C)** Endoscopic view of the spinal canal, confirming decompression of the gas-filled cavity and intact nerve root.

The patient was placed in the prone position, and the surgical field was sterilized and draped. A puncture site was marked 1 cm lateral to the L5/S1 spinous process. Under fluoroscopic guidance, a pencil-tip dilator was advanced to the inferior edge of the L5 lamina, followed by insertion of an outer cannula trephine to secure the lamina. A working channel was established, and a trephine was endoscopically advanced to loosen and remove the bony fragment. The ligamentum flavum was excised using a rongeur, resulting in immediate gas egress. Endoscopic exploration confirmed an intact annulus fibrosus, with no compression of the thecal sac or L5 nerve root exit zone. Absence of active bleeding was confirmed (Figure 4), and the procedure was concluded.

**Figure 5:**
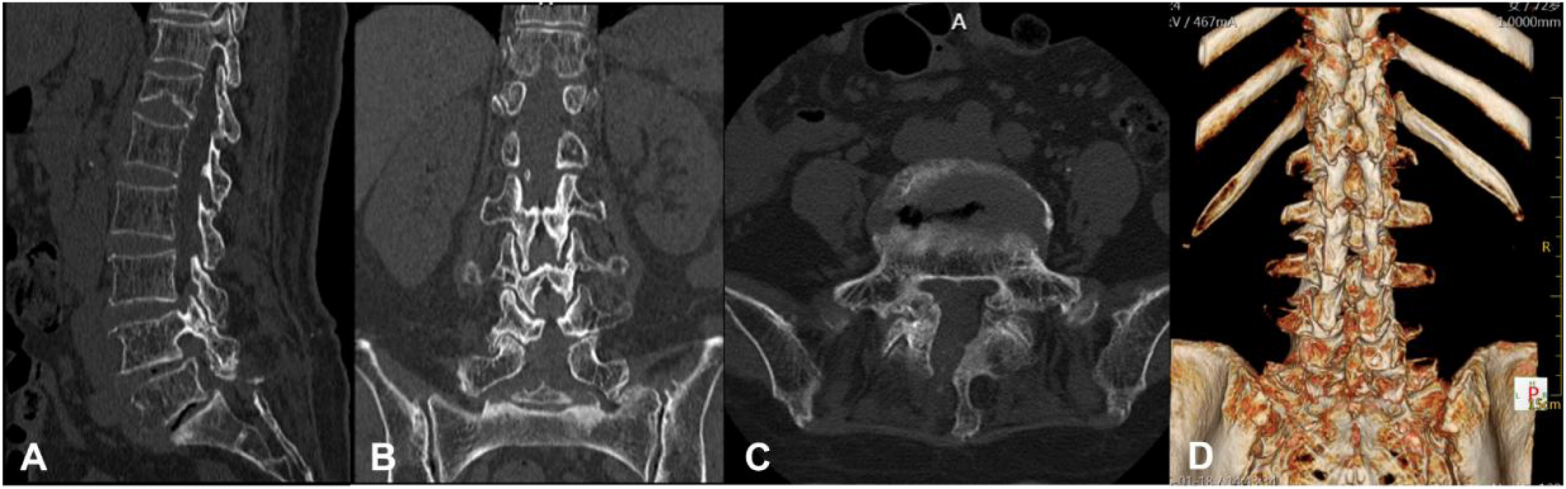
Postoperative lumbar three-dimensional CT scans. **(A)** Sagittal view demonstrates complete resolution of gas accumulation at the L4/5 and L5/S1 disc spaces and the posterior margin of the L5 vertebral body. **(B)** Coronal view shows absence of gas within the right L5 neural foramen. **(C)** Axial view confirms gas clearance in the right L5 lateral recess. **(D)** Three-dimensional CT reconstruction reveals a postoperative bony defect in the right L5 lamina following endoscopic decompression.

Postoperatively, the patient reported resolution of low back pain (VAS score for back from 5 to 0 within 24 h) and significant reduction in right leg radiculopathy (VAS score for the leg decreased from 6 to 2 within 24 h). She ambulated with a lumbar brace on postoperative day 1 and was discharged on day 3. Follow-up CT demonstrated complete resolution of gas in the right L5/S1 lateral recess, restored neuroforaminal volume, and an 8 mm surgical defect in the right lamina (Figures 5A–D). The VAS score for back remained at 0 at 3 months postoperatively, while the VAS score for the leg decreased to 1.

## 3 Discussion

Lumbar CT is considered the preferred imaging modality for diagnosing intradiscal gas accumulation due to its superior ability to delineate gas density (hypodense regions with CT values ranging from −900 to −1,000 Hounsfield units) and spatial distribution (1, 4, 6, 7). In the present case, CT clearly demonstrated gas migration into the right intervertebral foramen, providing critical anatomical guidance for surgical trajectory planning. Magnetic resonance imaging (MRI) complements CT by dynamically assessing nerve root compression through multi-sequence protocols (8). On T2WI, the hypointense gas pockets sharply contrast with adjacent hyperintense nerve root edema. However, fluid-attenuated inversion recovery (FLAIR) sequences should be interpreted with caution to avoid misdiagnosing gas signals as cerebrospinal fluid leakage. Conventional radiography, while capable of partially visualizing the vacuum disc phenomena, fails to directly demonstrate gas-induced compression of the dural sac or nerve roots. Notably, when hypointense signals are observed across T1-weighted imaging (T1WI), T2WI, and FLAIR sequences in lumbar MRI, CT remains essential to differentiate between dehydrated nucleus pulposus, gas accumulation, and calcified bone tissue.

Previous studies on the lumbar intervertebral vacuum phenomenon have primarily involved cases of lumbar spondylolisthesis or lumbar disc herniation, with surgical treatment mainly focusing on degenerative lumbar diseases. Research has found that patients with lumbar spondylolisthesis or disc herniation accompanied by the vacuum phenomenon tend to have poorer postoperative outcomes compared to those without the vacuum phenomenon (9). In their study, Ki et al. noted that lumbar spondylolisthesis patients undergoing posterior lumbar interbody fusion (PLIF) or posterolateral fusion (PLF) surgery showed no significant differences in clinical outcomes or fusion rates, regardless of the presence of the vacuum phenomenon (10). Conversely, Chang et al. reported a case of lumbar disc herniation with a vacuum phenomenon, which was successfully managed by removing the gas-containing herniated disc (11). The following table summarizes the diagnostic methods, concomitant diseases, treatment approaches, and therapeutic outcomes in some cases of lumbar intervertebral disc vacuum phenomenon (Table 1).

**Table 1:** Summary of clinical cases.

Case	Diagnostic methods	Treatment approaches	Therapeutic outcomes
Giyas Ayberk et al. (20)	MRI	Left L5-S1 hemilaminectomy and foraminotomy	Pain free
Yu Chen et al. (21)	MRI + CT	Percutaneous endoscopic lumbar nerve decompression surgery	Pain relieved
Mehdi Sasani et al. (22)	MRI + CT	Conservative treatment	Pain relieved
Kevin I Pak et al. (23)	MRI + CT	Percutaneous intradiscal aspiration of the vacuum disc gas	Pain relieved
Kyeong-Sik Ryu et al. (24)	MRI + CT	Microscopic discectomy	Pain completed relief

The patient's symptoms in this case were refractory to conservative management at an external institution, likely due to persistent nerve root compression by the gas-filled cavity and associated inflammatory mediator release (12), thus necessitating surgical intervention to alleviate mechanical compression (13). For patients with intractable pain, neurological deficits, or failed conservative therapy, percutaneous endoscopic interlaminar discectomy have emerged as the preferred treatment modality because of their minimally invasive nature and high efficacy (14). The primary advantage of percutaneous endoscopic decompression is its precision-targeted decompression capability (15). A posterior approach using a trephine allows for precise removal of lamina bone to access the ligamentum flavum, which can then be excised endoscopically to directly visualize and address the intraspinal gas-filled cavity and degenerated nucleus pulposus. In this case, immediate gas egress and restored nerve root pulsatility following ligamentum flavum excision confirmed the effectiveness of the decompression procedure. Compared to traditional open surgery, which involves extensive paraspinal muscle dissection, this technique utilizes only a 7.5 mm working channel with intraoperative blood loss of less than 10 ml.

While multiple surgical approaches exist for managing symptomatic VDP, percutaneous endoscopic decompression demonstrates unique advantages over conventional methods. Traditional open laminectomy requires extensive muscle dissection and laminectomy, increasing risks of postoperative instability (15%–20% complication rate) and prolonged recovery (16). Compared to conventional open lumbar discectomy, which necessitates larger incisions (6–8 cm) and results in higher bedtime (18–23 h) (17, 18), the endoscopic approach minimizes tissue trauma and accelerates functional recovery. Percutaneous endoscopic transforaminal discectomy (PETD), though minimally invasive, often fails to adequately address central or foraminal gas compression due to limited visualization. In contrast, PEID enable direct visualization of the lateral recess and neural foramen, facilitating complete gas evacuation (14). Moreover, Xian et al. demonstrated that both Unilateral Biportal Endoscopic Discectomy (UBED) and PEID exhibit excellent early therapeutic outcomes, but PEID offering the advantages of minimal invasiveness, familiar anatomy, and rapid puncture localization. The patient was able to ambulate with lumbar orthosis 4 h postoperatively, demonstrating >60% reduction in VAS pain scores within the first 24 h. Discharge occurred on postoperative day 3 with a return to daily activities, demonstrating the significant improvement in quality of life brought by this technique.

Although the PEID technique offers advantages such as minimal invasiveness and rapid recovery in treating VDP, it still presents several drawbacks. First, the procedure demands considerable surgical expertise, featuring a steep learning curve that may require novice surgeons to undergo extensive training before achieving proficiency. Second, its decompression efficacy may be limited when addressing severely calcified disc herniations or cases with significant lumbar instability. Additionally, the relatively narrow surgical field of view can pose challenges in controlling unexpected bleeding, such as from the epidural venous plexus, compared to UBE. Furthermore, for far-lateral disc herniations or severe foraminal stenosis, PEID may be less advantageous than the transforaminal endoscopic (PETD) approach.

## 4 Conclusions

In summary, the diagnosis of lumbosacral radiculopathy secondary to nerve root compression by intradiscal gas (vacuum disc phenomena) necessitates integrated multimodal imaging evaluation. Lumbar radiography serves as an initial screening tool to detect intravertebral gas collections. CT provides high-resolution spatial mapping of gas distribution and associated osseous abnormalities (e.g., spondylolysis, foraminal stenosis). MRI complements these modalities by dynamically assessing nerve root compression severity and perineural inflammatory responses through multi-sequence protocols, including T2WI and FLAIR. This triphasic imaging strategy minimizes diagnostic uncertainty and optimizes therapeutic decision-making. For patients with acute radiculopathy refractory to conservative therapy, PEID is advocated as the first-line intervention, offering distinct advantages over conventional approaches.

In the future, it is necessary to establish a clinical workflow for preoperative planning (such as gas volume threshold and compression anatomical localization), surgical treatment, and postoperative imaging verification of the vacuum phenomenon in intervertebral discs (19), and to conduct multicenter extended follow-up studies to validate long-term efficacy.

Furthermore, the pathogenesis of the intervertebral vacuum phenomenon remains unclear. Future studies incorporating molecular biology approaches are needed to determine whether the clinical benefits of PEID stem solely from mechanical decompression or also involve anti-inflammatory effects.

## Data Availability

The original contributions presented in the study are included in the article/Supplementary Material, further inquiries can be directed to the corresponding author.
